# The study on the relationship between perceived value, satisfaction, and tourist loyalty at industrial heritage sites

**DOI:** 10.1016/j.heliyon.2024.e37184

**Published:** 2024-08-29

**Authors:** Nengjie Qiu, Haibo Li, Chen Pan, Jiawei Wu, Jiaming Guo

**Affiliations:** a Architecture and Civil Engineering Institute, Guangdong University of Petrochemical Technology, Maoming, 525000, China; b Faculty of Innovation and Design, City University of Macau, Macau, 999078, China; c Visual Art and Desgin, Suan Sunandha Rajabhat University, Bangkok, 10700, Thailand

**Keywords:** Industrial heritage sites, Perceived value, Satisfaction, Loyalty, Open-pit mine ecological park

## Abstract

The legacy of industry reflects a city's journey and marks its cultural essence, highlighting the significance of preserving and promoting such historical assets. There is a noticeable gap in the discourse surrounding the intrinsic value of industrial heritage attractions and their impact on the fidelity of tourists. In light of this, the present investigation, rooted in the ABC attitude framework, examines the linkage between the perceived value, contentment, and allegiance of initial visitors to these sites. The study delves into the intermediary function of contentment and the variable influence of gender. Focusing on the Maoming Open-pit Ecological Park as a case in point, this research gathered and scrutinized 320 valid questionnaires. The outcomes demonstrate that the perceived value exerts a positive effect on both the satisfaction and the steadfastness of tourists. Satisfaction is identified as an intermediary between perceived value and tourist steadfastness. Additionally, gender plays a notable role in moderating specific aspects of the model. This inquiry aids in harnessing the potential of industrial heritage sites for the advancement of tourism, fostering economic prosperity and the rebirth of cultural practices. Furthermore, it lays down essential theoretical and practical building blocks for the evolution of industrial heritage as a tourism asset.

## 1 Introduction

The growth of tourism centered around industrial heritage presents a distinctive economic trend, embodying the essence of urban culture and documenting the evolution and advancement of metropolitan areas. The strategic deployment of these tourism resources is instrumental in safeguarding and highlighting the distinctive cultural heritage of a country, subsequently bolstering its cultural influence on the global stage. This form of tourism, which involves visiting sites to gain insights into historical operations, assess current scenarios, and anticipate future trends [1], appeals to a broad spectrum of travelers—those in search of authentic experiences and those seeking educational and contemplative journeys [2]. The appeal of industrial heritage tourism has been on the rise, offering visitors both educational and immersive experiences. The cultivation of such tourism resources aids in the harmonious integration of industrial spaces within urban settings, optimizes urban functional planning, and fosters the growth of this niche tourism sector, which is pivotal in conserving the legacy of urban industrial practices [3].

The propensity of travelers to return and recommend a destination is closely linked to the concept of perceived value, which encompasses a person's comprehensive assessment of the quality and services offered at a particular site [4]. Within the realm of consumer behavior, perceived value is recognized as a robust indicator of visitor contentment and allegiance [5], contingent upon the evaluation of information, service excellence, tourism assets, as well as the time, financial, and energetic investments made in the visit. Given that diverse tourism venues provide distinct offerings, there is an ongoing academic discourse regarding the precise meaning of perceived value. Prior investigations into visitor loyalty have predominantly concentrated on the quality of the destination, perceived value, local bond, and the depth of tourism engagement [[6], [7], [8], [9]], with a relative dearth of systematic examinations of how perceived value at a destination influences loyalty. Consequently, delving into the role of perceived value in fostering tourist loyalty at industrial heritage sites is of considerable theoretical and applied importance. Moreover, gender, as a demographic element, highlights individual differences in tourist traits and emotional responses. Presently, the majority of gender-based research is conducted at a descriptive statistical level, with a limited exploration of gender's potential as a moderating influence within research frameworks.

This inquiry, carried out via a survey at the Maoming Open-pit Mine Ecological Park and anchored in the ABC attitude model, scrutinizes the influence of perceived value components on tourist loyalty, with visitor satisfaction acting as an intermediary. Additionally, gender is incorporated as a moderating factor to further investigate its role within the model. The objective of this research is to deepen the comprehension of tourist loyalty at industrial heritage locales and to offer insights for the more effective development and dissemination of strategies pertaining to industrial heritage tourism.

This study, conducted through a questionnaire survey at Maoming Open-pit Mine Ecological Park, is based on the ABC attitude model. It examines the impact mechanism of perceived value dimensions on tourist loyalty, with tourist satisfaction serving as a mediating variable. Furthermore, gender is considered as a moderating variable to further explore its effects within the research model. This study aims to enhance the understanding of tourist loyalty at industrial heritage destinations, providing reference value for better formulation and promotion of industrial heritage tourism strategies.

## 2 Literature review and theoretical foundations

### 2.1 Perceived value

The idea of perceived value, which originated in marketing, serves as a crucial tool for predicting and deciphering consumer choices and purchasing behaviors [10]. Initial studies suggested that perceived value is made up of the perceived benefits like economic, social, and relational gains, as well as the sacrifices such as price, time, effort, risk, and convenience that customers make [11,12]. In the context of tourism, perceived value is the evaluation by an individual of a tourism product, taking into account factors like service quality, pricing, emotional connection, and social elements [13]. This concept has been explored across different tourism types, including adventure, rural, and eco-tourism [[14], [15], [16]]. Perceived value can be measured in either a one-dimensional approach or a multi-dimensional scale [17]. The one-dimensional approach assumes a uniform consumer perception of value [18], while the multi-dimensional approach offers a more detailed view of the various components that constitute perceived value. Kim and Park [19] introduced an all-encompassing model of perceived value that includes economic, functional, emotional, and social aspects. Expanding on this, Kim and Thapa [20] identified perceived value to be composed of four key dimensions: quality, emotional response, cost, and social impact. In addition, Carvacho-Franco et al. [5].proposed that perceived value comprises three main dimensions: economic, functional, and emotional/social. Presently, there is a gap in the evaluation of perceived value within the realm of industrial heritage tourism. Further research is warranted to identify the dimensions of perceived value that resonate with tourists in the context of industrial heritage sites.

### 2.2 Tourist satisfaction

Achieving tourist contentment is a key objective for service-oriented organizations, as it tends to result in higher revisit rates and favorable. Satisfaction can be described as "an assessment that the features of a product or service, or the experience itself, delivers an enjoyable consumption experience" [21]. The concept of consumer satisfaction extends beyond cognitive appraisal, encompassing emotional aspects as well [22]. Present inquiries into satisfaction generally focus on two categories: immediate (specific to a transaction) and general (an aggregate of experiences). Immediate satisfaction pertains to the assessment of a singular interaction or occurrence within a service context. On the other hand, general satisfaction reflects a comprehensive evaluation of the entire service interaction, drawing from all encounters with the service provider [23]. While immediate satisfaction can fluctuate based on personal experiences, general satisfaction is considered to be more consistent over time [24]. In this vein, the current investigation aligns with Oliver's stance, treating satisfaction as the aggregate emotional reaction to the overall service encounter, particularly during a tourist's inaugural visit to an industrial heritage site.

### 2.3 Tourist loyalty

Loyalty has garnered significant attention in the field of service marketing because it creates a sustainable competitive advantage for service organizations [25]. As such, service managers should tailor their marketing strategies to maintain customer loyalty, taking into account their specific circumstances. In the realm of consumer behavior, loyalty is defined as a behavioral commitment to purchase products or services in the future [26], or the steadfast behavior of re-purchase and patronage despite potential market changes and marketing efforts that could lead to switching [27]. Destination loyalty is typically assessed through tourists' behavioral intentions, which are measured by their willingness to revisit the destination and their intention to spread positive word-of-mouth and make recommendations [28].

### 2.4 Theoretical foundations

The ABC model, recognized for its "Cognition-Affect-Behavior" framework, was introduced by Rosenberg in 1960, focusing on the influence of environmental contexts on consumer actions [29]. This model suggests that behaviors are driven by expectations linked to specific activities. Cognition serves as the base for both affect and behavior, with affect acting as a mediator between cognition and behavior, leading to behavior being shaped by both cognitive elements and emotional influences.

Research has shown that the ABC attitude model effectively elucidates decision-making in tourism, with successful applications in fields like forest wellness tourism [30]and sustainable travel practices [31].

## 3 Research hypotheses and model proposition

Based on the ABC attitude model, we consider tourists' perceived value as the cognitive component (A), tourists' satisfaction as the affective component (B), and tourists' loyalty as the behavioral component (C). Using this theoretical framework and research hypotheses, we construct a structural relationship model.

### 3.1 The relationship between perceived value and satisfaction

Perceived value is considered a predictor of tourist satisfaction. The higher the perceived value of the overall quality and service of the destination, the higher the tourist satisfaction with the attraction. Frewer found that an individual's cognition towards a tourist destination can elicit emotional responses [32]. Zhu Xiaoqing and Sui Wenjing conducted an empirical analysis of Xi'an Heritage Theme Park, confirming that perceived value significantly influences tourist satisfaction [33]. Huang Xijia et al., using theme parks as an example, discovered a positive correlation between perceived value and tourist satisfaction [34]. Additionally, Matsuoka et al., studying Japan's Salt Village, found that the perceived value of local restaurants, ambiance, and souvenirs had a significant positive impact on tourist satisfaction [35]. Accordingly, the following hypothesis is proposed.

H1a Economic value has a positive impact on tourist satisfaction.

H1b Functional value has a positive impact on tourist satisfaction.

H1c Emotional value has a positive impact on tourist satisfaction.

H1d Social value has a positive impact on tourist satisfaction.

### 3.2 The relationship between perceived value and loyalty

Existing literature suggests that there is a correlation between perceived value and the loyalty of tourists, with perceived value having a favorable impact on loyalty [36]. A study by Peña et al., which investigated the dynamics between perceived value and loyalty in rural tourism in Spain, concluded that perceived value positively affects loyalty [37]. Gallarza et al. applied the LISREL model to examine the travel tendencies of university students and determined that perceived value plays a substantial role in shaping loyalty [38]. In alignment with these findings, Jin et al.'s investigation into theme park attendance in North America revealed that perceived value strengthens tourists' propensity for recommendations and repeat visits [39]. In light of these insights, the following hypotheses can be put forward.

H2a Economic value positively affects tourist loyalty.

H2b Functional value positively affects tourist loyalty.

H2c Emotional value positively affects tourist loyalty.

H2d Social value positively affects tourist loyalty.

### 3.3 The relationship between satisfaction and loyalty

Within the realm of travel and destination promotion, a consensus among many academics is that satisfaction plays a pivotal role in shaping tourist loyalty [40]. Tourists who have a satisfying experience at an attraction or destination are more inclined to consider returning and are also prone to share their favorable experiences through positive recommendations [41]. A study conducted by Cheng et al., focusing on the Song City Historical and Cultural Theme Park in Hangzhou, China, affirmed the profound influence of theme park satisfaction on fostering loyalty [42]. Echoing this, Milman et al. observed a similar positive relationship between satisfaction and loyalty among theme park patrons in North America [43]. Moreover, Ali et al., in their examination of emotional encounters of tourists at Malaysian theme parks, discovered that satisfaction exerts a beneficial impact on enhancing loyalty [44].

H3 Satisfaction has a positive influence on tourist loyalty.

### 3.4 The mediating role of satisfaction

The ABC attitude model indicates that cognition refers to an individual's perception of the destination, affect pertains to an individual's feelings towards the destination, and behavior represents the individual's behavioral intentions towards the destination.The discourse has illustrated that perceived value exerts a favorable and notable influence on tourist contentment, with satisfaction subsequently acting as a key driver of tourist loyalty [45,46]. Meanwhile, satisfaction has been proven to mediate the relationship between tourists' perceptions and loyalty [47]. Zhao Lei et al.'s research found that perceived value positively affects tourist loyalty and indirectly influences it through satisfaction. In national wetland parks, tourists develop various dimensions of perceived value through their interactions with the destination. They weigh the pros and cons of local customs, cultural connotations, and financial expenditures. When tourists' perceived value is met, leading to positive emotional responses, they exhibit higher satisfaction. This satisfaction enhances their impression of the destination and fosters a willingness to recommend it to family and friends, as well as a desire to revisit. Hence, satisfaction acts as a bridge between perceived value and tourist loyalty [48]. Accordingly, the following hypothesis is proposed.

H4 Satisfaction has a mediating role between perceived value and tourist loyalty.

### 3.5 The moderating effect of gender

The encounter of visitors at a site or attraction is a mental occurrence that manifests through emotional responses, signifying an enjoyable interaction [49]. Within the scope of psychological studies, variations in gender can result in notable disparities in the perceptions and experiences of tourists at a destination, with distinct emphases and emotional interpretations [50]. Bendall-Lyon et al. have observed clear distinctions in the way gender influences perception and loyalty among travelers [51]. Moreover, Han and Ryu have highlighted the moderating influence of gender on the relationship between satisfaction and loyalty [52]. Consequently, gender-based differences may shape the way tourists assess the attributes and services of industrial heritage tourism, along with the interplay among perceived value, satisfaction, and loyalty. In light of these considerations, the following hypotheses can be postulated.

H51a Gender has a significant moderating effect on the relationship between economic value and tourist satisfaction.

H51b Gender has a significant moderating effect on the relationship between functional value and tourist satisfaction.

H51c Gender has a significant moderating effect on the relationship between emotional value and tourist satisfaction.

H51d Gender has a significant moderating effect on the relationship between social value and tourist satisfaction.

H52a Gender has a significant moderating effect on the relationship between economic value and tourist loyalty.

H52b Gender has a significant moderating effect on the relationship between functional value and tourist loyalty.

H52c Gender has a significant moderating effect on the relationship between emotional value and tourist loyalty.

H52d Gender has a significant moderating effect on the relationship between social value and tourist loyalty.

H53 Gender has a significant moderating effect on the relationship between satisfaction and tourist loyalty.

**Fig. 1:**
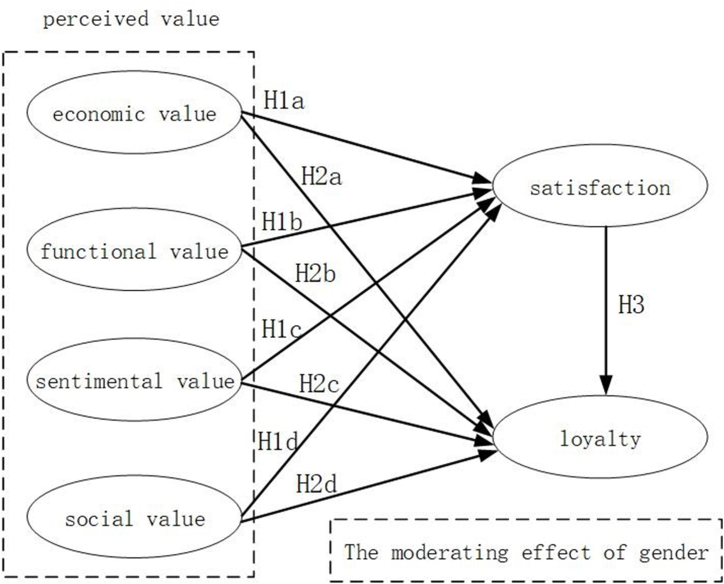
Research model.

To encapsulate the findings, this research, grounded in the ABC attitude framework, critical literature, and formulated hypotheses, delves into the motivational underpinnings of how perceived value influences tourist loyalty within the context of industrial heritage sites. In this model, satisfaction operates as the intermediary variable, while gender is considered as the variable that adjusts the impact (refer to Fig. 1).

## 4 Research design

### 4.1 Overview of the case study site

**Fig. 2:**
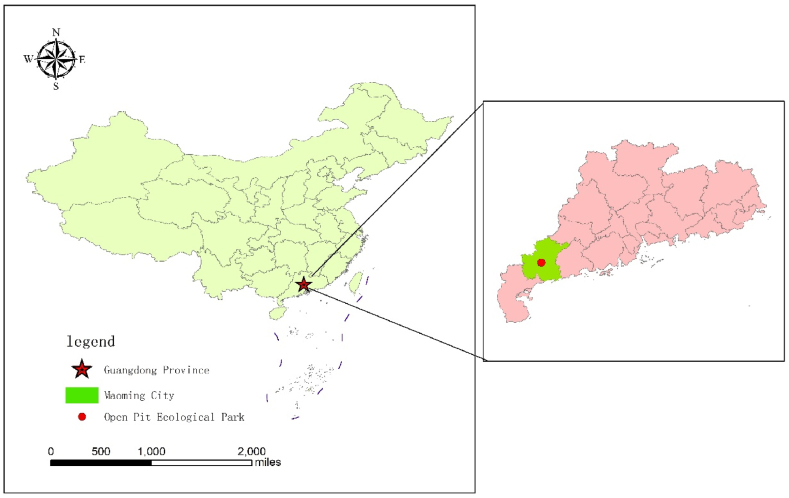
Maoming open-pit mine ecological park.

Maoming Open-pit Mine Ecological Park is situated in the Maonan District of Maoming City (Fig. 2). It is the second-largest open-pit mine in China, a legacy of the early oil extraction process through shale retorting. The park spans an area of 10.07 square kilometers, rich in mineral resources, and represents a typical industrial and mining heritage site. Beyond the pit lake, the ecological park features distinctive German-style wooden cabins, a sea of wind chime flowers, wetland corridors, and leisure attractions such as steam locomotives and "Jiefang" brand trucks stationed on the railway tracks. The park also encompasses facilities like a museum and a children's playground, which not only serve visitors but also add to the park's diversity. Additionally, the park regularly hosts a variety of educational programs, including ecological restoration and greening of mine sites, revitalization of abandoned pits, and the promotion of mining culture and the spirit of the oil city.

In 2017, Maoming Open-pit Mine Ecological Park was designated as a "National Mine Park," and by 2023, it was recognized as a model environmental education base in Guangdong Province and included in the "Seventh Batch of 20th Century Chinese Architectural Heritage" projects. Therefore, surveying tourists at this park can offer academic and managerial insights for similar types of tourism nationwide.

### 4.2 Questionnaire design

For the purpose of data collection, a research survey instrument was crafted. The survey is divided into two distinct segments. The initial segment gathers information on the demographic traits of first-time tourists. The second segment introduces six distinct measurement variables: economic value, functional value, emotional value, social value, satisfaction, and loyalty. The items used to assess these constructs are sourced from established research scales known for their dependability and credibility.

The survey employs a standard 7-point Likert-type scale for responses, with the anchor of 1 indicating strong disagreement and 7 indicating strong agreement. Perceived value is further categorized into four sub-dimensions: economic (assessed by 3 items), functional (4 items), emotional (4 items), and social value (4 items). These measurement items are informed by the studies of Kim and Park [19], Hapsari [53], Carvache [5], among others. Satisfaction is operationalized through 3 items, drawing from the work of Chen [54] and Yoon and Uysal [55]. Lastly, the loyalty construct is gauged by 3 items, which are taken from the research of Pike [56] and Ha and Jang [57].

### 4.3 Data collection

On October 15, 2022, the survey team conducted a pre-test by selecting 50 first-time visitors to the open-pit mine ecological park. They also sought opinions from five experts in the tourism field and destination managers. Based on the results of the preliminary research, the questionnaire was adjusted and modified. For the main survey, random intercept interviews were conducted at key locations such as park entrances, museums, fitness squares, parking lots, children's play areas, and wetland corridors. These areas are not only entry points to the park but also high-traffic zones where visitors gather and relax, thus improving the representativeness and accuracy of the sample.To safeguard the integrity of the data gathered, all researchers participated in standardized training sessions before initiating the distribution of the questionnaires. This process ensured that they fully comprehended the study's goals and protocols. The formal data collection took place from November 5 to December 15, 2022. The survey sample had to meet two criteria: respondents had to be at least 18 years old and visiting the destination for the first time. Eligible respondents were then asked to indicate the extent to which they agreed with the statements in the questionnaire and how these statements related to their current situation. In this study, 350 questionnaires were distributed, and after excluding incomplete responses, 320 valid questionnaires were obtained, resulting in a response rate of 97.2 %.

Throughout the study, a total of 350 questionnaires were made available to potential respondents. After the removal of any incomplete or inadequate responses, a total of 320 valid responses were collected, representing an effective response rate of 91.7 %.

## 5 Results

### 5.1 Basic characteristics of respondents

Table 1 indicates the demographic distribution of the participants in this study. The gender distribution is relatively balanced, with females comprising 45.9 % and males 54.1 % of the respondents. The majority of the participants fall within the age range of 18–35 years, totaling 178 individuals, which is 55.7 % of the sample. The 36–45 years age bracket accounts for 21.3 % of the participants. In terms of education, a minority, 29.7 %, have completed their education at the high school or technical secondary school level, while the remaining 70.3 % have attained a junior college degree or higher. Regarding employment, approximately a fourth of the participants, 26.6 %, are employed in enterprises or corporations. Concerning income levels, 106 individuals, which is 33.1 % of the respondents, report earning between 2001 and 5000 yuan.

**Table 1:** Basic information of respondents.

Variable	Description	Frequency	Percentage
Gender	Male	173	54.1 %
	Female	147	45.9 %
Age	18–22 years old	52	16.3 %
	23–35 years old	126	39.4 %
	36–45 years old	68	21.3 %
	46–55 years old	43	13.4 %
	56 years old and above	31	9.7 %
Educational Attainment	High school and vocational school	95	29.7 %
	College and undergraduate	184	57.5 %
	Postgraduate and above	41	12.8 %
Occupation	Government and public institution employees	57	17.8 %
	Enterprise/Company employees	85	26.6 %
	Students	55	17.2 %
	Freelancers	72	22.5 %
	Retired employees	18	5.6 %
	Other	33	10.3 %
Income Situation	Below 2000 yuan	34	10.6 %
	2001-5000 yuan	106	33.1 %
	5001-8000 yuan	76	23.8 %
	8001-10000 yuan	59	18.4 %
	Above 10000 yuan	45	14.1 %

### 5.2 Reliability and validity analysis

In the initial phase of the factor analysis, an item stating "I receive recognition from others" was identified to have a factor loading below the recommended threshold of 0.6, leading to its exclusion. The remaining factor analysis confirmed that perceived value is structured around four distinct dimensions, which were named economic value, functional value, emotional value, and social value, based on the content of the items.Subsequently, to evaluate the measurement model's fit, as well as its reliability and validity, a confirmatory factor analysis was performed using Amos 24.0.

The fit indices yielded satisfactory results (χ2/df = 2.376, GFI = 0.923, CFI = 0.937, TLI = 0.952, NFI = 0.949, AGFI = 0.951), indicating a well-fitted model. As presented in Table 2, the Cronbach's α values for the dimensions of perceived value, satisfaction, and loyalty ranged from 0.805 to 0.904, exceeding the minimum acceptable level of 0.7. The composite reliability (CR) scores also fell between 0.762 and 0.906, which is above the recommended cut-off of 0.6. Moreover, the average variance extracted (AVE) values spanned from 0.517 to 0.764, all surpassing the 0.5 benchmark. These findings suggest that the latent variables possess adequate reliability and convergent validity [58,59].

**Table 2:** Confirmatory factor analysis table.

Variable and Measurement Items	Factor Loading	CR	AVE	Cronbach's α
**Economic Value**		0.906	0.764	0.904
The destination is economical	0.791			
The transportation costs at the destination are reasonable	0.954			
The prices of souvenirs and food at the destination are reasonable	0.869			
**Functional Value**		0.89	0.671	0.890
The destination is convenient for me	0.864			
The overall layout of the destination is well arranged	0.789			
The destination provides a lot of information related to mining culture	0.773			
The directional signs are conspicuous and easy to find related attractions	0.846			
**Emotional Value**		0.823	0.538	0.821
The destination makes people feel relaxed	0.706			
The destination makes people feel happy	0.742			
The destination generates positive feelings	0.79			
The destination enhances relationships with family or friends	0.692			
**Social Value**		0.812	0.531	0.820
This trip allows me to make more friends	0.877			
This trip leaves a good impression on others	0.843			
This trip provides a novel experience	0.624			
This trip inspires me to learn more about industrial heritage culture	0.504			
**Satisfaction**		0.762	0.517	0.805
I think this trip is the right choice	0.771			
All my expectations for this trip have been met	0.685			
I think the time and effort spent on this trip are worthwhile	0.69			
**Loyalty**		0.848	0.653	0.868
I am willing to visit the destination again	0.774			
I would recommend the destination to family or friends	0.924			
I will promote positive information about the destination to others	0.712			

The concept of discriminant validity, which is crucial for assessing the distinctiveness of various variables, is deemed optimal when the square root of the Average Variance Extracted (AVE) surpasses the inter-correlations of the other variables, as supported by O'Leary-Kelly et al. [60]. As depicted in Table 3, the square root of AVE for each of the six latent variables exceeds the inter-correlations with other variables, confirming robust discriminant validity for all latent constructs in the study.

**Table 3:** Discriminant validity table.

Variable	AVE	Economic Value	Functional Value	Emotional Value	Social Value	Satisfaction	Loyalty
Economic Value	0.764	**0.874**					
Functional Value	0.671	0.11	**0.819**				
Emotional Value	0.538	0.397	0.529	**0.733**			
Social Value	0.531	0.190	0.728	0.719	**0.729**		
Satisfaction	0.517	0.387	0.573	0.706	0.709	**0.719**	
Loyalty	0.653	0.434	0.493	0.731	0.615	0.707	**0.808**

### 5.3 Hypothesis testing

**Fig. 3:**
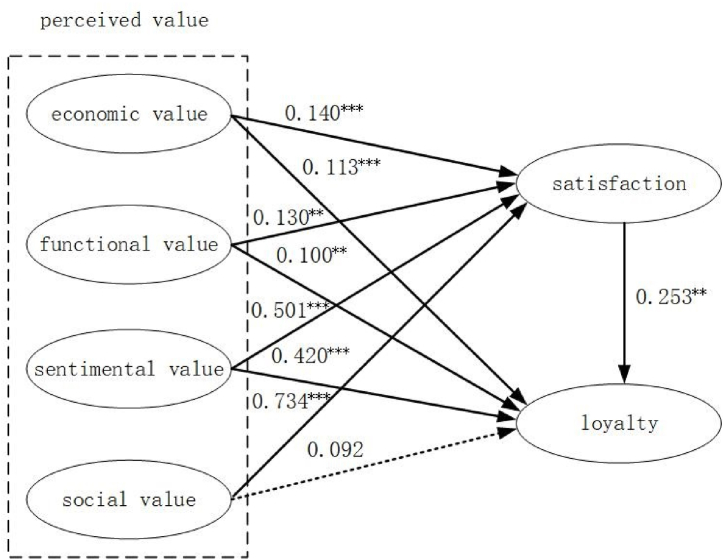
Statistical model.

The structural equation modeling (SEM) approach was employed to evaluate the proposed research hypotheses, with the findings depicted in Fig. 3 and Table 4. The path coefficients for hypotheses H1a, H1b, H1c, and H1d, which are associated with economic, functional, emotional, and social values, are 0.140 (P < 0.001), 0.130 (P < 0.01), 0.501 (P < 0.001), and 0.734 (P < 0.001), respectively. These results suggest that all four types of value positively and significantly influence tourist satisfaction, providing support for hypotheses H1a, H1b, H1c, and H1d. In other words, enhancing tourists' recognition of the economic, functional, emotional, and social values of a destination can improve their satisfaction levels. Furthermore, among the four dimensions of perceived value that influence satisfaction, the social value has the greatest impact (0.734). Therefore, understanding the reasons behind tourists' cognition of social value in detail will be beneficial in formulating suggestions to enhance visitor satisfaction. The standardized path coefficients for H2a, H2b, H2c, and H2d are 0.113 (p < 0.001), 0.100 (p < 0.01), 0.420 (p < 0.001), and 0.092 (p > 0.05), respectively. Therefore, Hypothesis H2d is not supported, indicating that there is no significant direct association between social value and tourist loyalty. The remaining three hypotheses are all supported. It is evident that enhancing tourists' perception and recognition of economic, functional, and emotional values during their travel experience can influence their subsequent behavioral intentions. Moreover, among the four dimensions of perceived value that affect loyalty, emotional value has the greatest impact (0.420). Therefore, gaining a comprehensive understanding of tourists' acknowledgment of emotional value is crucial for devising strategies to strengthen their loyalty. The standardized path coefficient for H3 is 0.253 (p < 0.01), indicating that satisfaction has a positive effect on tourist loyalty. Hypothesis H3 is also supported. Therefore, enhancing visitor satisfaction is the top priority for strengthening the loyalty of tourists to a destination.

**Table 4:** Results of hypothesis testing.

Hypothetical Path	Estimate	S.E.	C.R.	P-value	Conclusion
H1a: Economic Value → Satisfaction	0.140	0.032	4.375	***	Supported
H1b: Functional Value → Satisfaction	0.130	0.046	2.826	0.004	Supported
H1c: Emotional Value → Satisfaction	0.501	0.076	6.592	***	Supported
H1d: Social Value → Satisfaction	0.734	0.113	6.496	***	Supported
H2a: Economic Value → Loyalty	0.113	0.029	3.897	***	Supported
H2b: Functional Value → Loyalty	0.100	0.039	2.564	0.010	Supported
H2c: Emotional Value → Loyalty	0.420	0.080	5.250	***	Supported
H2d: Social Value → Loyalty	0.092	0.094	0.978	0.328	Not Supported
H3: Satisfaction → Loyalty	0.253	0.089	2.843	0.005	Supported

### 5.4 Mediation effect test

In this research, the non-parametric percentile Bootstrap technique, utilizing 5000 bootstrap samples, was applied to assess the mediating role of satisfaction in the relationship between perceived value and tourist loyalty, as well as to determine the overall, indirect, and direct impacts (refer to Table 5). The findings reveal that the confidence intervals for both the total and indirect effects of economic and emotional values on tourist loyalty do not encompass zero. This suggests that satisfaction mediates partially in the link between economic and emotional values and tourist loyalty. This indicates that among the four dimensions of perceived value, both economic value (0.113) and emotional value (0.420) not only have a direct impact on loyalty but also exert an indirect influence on loyalty through the mediating role of satisfaction. The indirect effects of economic and emotional values are 0.035 and 0.127, respectively. Among these, the emotional value has the greatest effect. Therefore, focusing on tourists' emotional responses during their destination visit can help increase their satisfaction and, in turn, enhance loyalty. Conversely, the confidence intervals for the direct effects of functional and social values on tourist loyalty include zero, while the intervals for the total and indirect effects are above zero. This points to a full mediating function of satisfaction in the connection between functional and social values and tourist loyalty. Consequently, hypothesis H4 is supported.

**Table 5:** Results of mediation effect test.

Hypothetical Path	Effect	Point Estimate Value	Bias-Corrected 95 % CI		Percentile 95 % CI		Conclusion
			Lower	Upper	Lower	Upper	
Economic Value → Satisfaction → Loyalty	Total Effect	0.148	0.07	0.243	0.063	0.225	Supported
	Indirect Effect	0.035	0.004	0.091	0.002	0.080	Supported
	Direct Effect	0.113	0.023	0.213	0.015	0.207	Supported
Functional Value → Satisfaction → Loyalty	Total Effect	0.133	0.020	0.236	0.023	0.241	Supported
	Indirect Effect	0.033	0.005	0.097	0.001	0.078	Supported
	Direct Effect	0.100	−0.016	0.206	−0.016	0.209	Not Supported
Emotional Value → Satisfaction → Loyalty	Total Effect	0.547	0.305	0.78	0.335	0.803	Supported
	Indirect Effect	0.127	0.014	0.292	0.006	0.284	Supported
	Direct Effect	0.420	0.184	0.663	0.209	0.688	Supported
Social Value → Satisfaction → Loyalty	Total Effect	0.278	0.061	0.562	0.048	0.545	Supported
	Indirect Effect	0.186	0.022	0.451	0.009	0.427	Supported
	Direct Effect	0.092	−0.145	0.369	−0.148	0.364	Not Supported

### 5.5 Moderating effect of gender

This investigation examined the influence of gender as a moderating variable by developing separate structural equation models for male and female participants. As indicated in Table 6, the findings demonstrate that gender plays a moderating role in the connections between economic value and emotional value with satisfaction, as well as between economic value and tourist loyalty, and satisfaction and tourist loyalty, thereby supporting hypotheses H51a, H51c, H52a, and H53. In these four relationships where gender acts as a moderator, the path coefficients were found to be significantly more pronounced in the male group than in the female group. Moreover, gender was observed not to have a significant moderating impact on the other relationships tested.

**Table 6:** Results of gender moderation effects.

Hypothetical Path	Female Group (Beta)	t-value	Male Group (Beta)	t-value	Coefficient Difference Critical Ratio	Conclusion
H51a:Economic Value→Satisfaction	0.164	4.362	0.457	6.956	−3.847	Supported
H51b:Functional Value→ Satisfaction	0.298	5.791	0.336	0.043	−0.563	Not Supported
H51c:Emotional Value→ Satisfaction	0.404	9.057	0.579	10.776	−2.489	Supported
H51d:Social Value→ Satisfaction	0.436	10.417	0.463	10.437	−0.444	Not Supported
H52a: Economic Value→ Loyalty	0.570	9.677	0.756	12.827	−2.229	Supported
H52b:Functional Value → Loyalty	0.287	5.301	0.283	6.487	0.057	Not Supported
H52c:Emotional Value→ Loyalty	0.476	10.827	0.537	10.204	−0.886	Not Supported
H52d:Social Value→ Loyalty	0.389	8.409	0.428	9.821	−0.613	Not Supported
H53:Satisfaction→ Loyalty	0.207	5.418	0.464	7.718	−3.617	Supported

## 6 Discussion and conclusion

### 6.1 Discussion

First, the impact of perceived value on tourists' satisfaction and loyalty. Empirical results show that economic value, functional value, emotional value, and social value have a positive impact on tourists' satisfaction. Similarly, economic value, functional value, and emotional value can significantly influence the formation of tourists' loyalty. This conclusion corroborates Li's [61] view that perceived value triggers tourists' loyalty, as well as Dai Qiwen and Guo Zijing's [62] proposition that perceived value plays an important driving role in tourists' satisfaction. It indicates that perceived value can significantly enhance tourists' satisfaction and loyalty. The difference lies in that the aforementioned studies examine perceived value as an overall dimension, while this study breaks down perceived value and considers its multidimensional perspective. The research findings can better indicate the direction for enhancing tourists' satisfaction and loyalty. It is worth noting that social value does not have a direct impact on tourists' loyalty, which is inconsistent with the findings of Carvache et al. [5]. The underlying reason, according to this study, is that social value reflects the process of interaction with people and understanding of the destination, which is a relatively long-term concept. It may be that the short duration of the first visit by tourists affects their perception of social value.

Second, the mediating role of satisfaction. The impact of economic value, functional value, emotional value, and social value on tourists' loyalty can all be realized through the mediation of tourists' satisfaction. The study shows that satisfaction plays a partial mediating role between economic value, emotional value, and tourists' loyalty, which is in line with the views of Ranjbarian and Pool [63], and Chen Zhijun and Xu Feixiong [64]; it plays a complete mediating role between functional value, social value, and tourists' loyalty. This is inconsistent with the views of scholars such as Guo Anxi [65], and the possible reason is that the functional value provided by industrial heritage destinations to first-time visitors only meets their temporary material needs. First-time visitors only have a brief and superficial tour of the destination, and many functional facilities have not been deeply perceived and used, thus failing to quickly generate loyalty. In addition, satisfaction plays a complete mediating role between social value and tourists' loyalty largely because the impact of the social value variable on tourists' loyalty is not significant. Moreover, Dai Qiwen et al. tested the mediating role of satisfaction between perceived value and tourists' loyalty in homestay tourism [66]. This has enriched the research content on exploring tourists' loyalty from individual perceived value, and is conducive to a comprehensive understanding of the mechanism from individual perceived value to the generation of loyalty.

Third, the moderating role of gender. Empirical results indicate that the relationships between economic value and satisfaction, emotional value and satisfaction, economic value and loyalty, and satisfaction and loyalty are influenced by gender, with male path coefficients being higher than those of females in predictive effects. Existing studies show that there are significant differences between men and women in terms of tourism experience and behavior [67]. This may be due to different emotional responses of men and women in the process of social interaction. Furthermore, Bendall-Lyon and Powers pointed out that consumers of different genders have significant differences in the formation process of satisfaction [68]. Weng et al. also pointed out that gender is an important factor affecting tourists' entertainment experience, environmental attitudes, and environmental protection behaviors [69]. Therefore, for destination managers, it is necessary to enhance the perceived value of female tourists to form a positive satisfaction and loyalty, thereby obtaining a satisfactory tourism experience.

### 6.2 Conclusion

This study found that, first, all four dimensions of perceived value have a significant positive impact on satisfaction, with the effect sizes ranked from largest to smallest as social value, emotional value, economic value, and functional value. Second, three dimensions of perceived value have a significant positive impact on tourists' loyalty, with the effect sizes ranked from largest to smallest as emotional value, economic value, and functional value, while social value does not have a significant impact on tourists' loyalty. Third, satisfaction acts as a mediator between perceived value and tourists' loyalty, where satisfaction partially mediates the impact of economic value and emotional value on tourists' loyalty, and fully mediates the impact of social value and functional value on tourists' loyalty. Fourth, gender plays a moderating role in the relationships between economic value and emotional value with satisfaction, economic value with tourists' loyalty, and satisfaction with tourists' loyalty, while other path relationships did not reach the range of significance.Thirdly, this study incorporates gender as a moderating variable, empirically testing its regulatory role in different paths of the conceptual model. This comprehensively reflects the impact mechanism of destination perceived value and satisfaction on tourist loyalty, laying the groundwork for future research in the tourism field that addresses differences between individual genders.

### 6.3 Theoretical significan

This study has made several theoretical contributions to the field of destination perceived value and tourist loyalty.

Firstly, previous research has primarily treated perceived value as a holistic construct, with less consideration given to the impact of economic value, functional value, emotional value, and social value on tourists' satisfaction and loyalty. This study adopts a multidimensional perspective of perceived value, resulting in more nuanced research conclusions compared to previous scholars.

Secondly, building upon previous studies, this research constructs a Perceived Value → Satisfaction → Loyalty framework using the ABC attitude model. Through empirical research, it examines the transmission mechanism of economic value, functional value, emotional value, and social value on tourists' loyalty via satisfaction. This helps explain how tourists develop loyalty to industrial heritage destinations through their experience of perceived value, enriching the theoretical understanding of the relationship between perceived value and tourist loyalty. This will aid in bringing more social and economic benefits to tourism destinations and laying the foundation for future research in this field.

Thirdly, this study incorporates gender as a moderating variable, empirically testing its moderating effects on different paths within the conceptual model. This comprehensively reflects the impact mechanism of destination perceived value and satisfaction on tourists' loyalty, laying the groundwork for future research in tourism that addresses the differences between individuals of different genders.

### 6.4 Managerial implications

This research offers several practical recommendations for the sustainable management of industrial heritage sites:

Firstly, the role of perceived value dimensions in enhancing tourist loyalty. Empirical studies have shown that emotional value, economic value, and functional value positively affect tourist loyalty, with emotional value being the most significant predictor of tourists' willingness to recommend and evaluate the industrial heritage destination. To this end, destination managers can take the following measures to enhance tourist loyalty. First, managers can improve the provision of activities related to emotional value, offering tourists new experiences or adventures. For example, using AR and VR technology to provide virtual tour spaces, allowing tourists to interact with virtual products in real-time, or using animated videos to restore the "coal washing" process and working scenes, educating tourists on the entire process from coal mining to formation, and creating an immersive experience atmosphere that evokes tourists' emotional resonance. Additionally, developing cultural and creative products with industrial symbols according to market demand, using unique symbolic language and cultural imprints to further resonate with tourists' emotions. Secondly, strengthen the management of destination products and service prices, clearly mark the prices for services such as entertainment, transportation, and catering in the destination, strictly control quality, and combat false practices to ensure the economic value of destination products and services. Lastly, managers should regularly replace or upgrade components that do not provide positive or memorable experiences based on market demand and actively improve destination infrastructure services to ensure the functional value of the services.

Secondly, it's important to recognize the influence of perceived value on satisfaction and to leverage satisfaction as a means to bolster tourist loyalty. Research indicates that economic, functional, emotional, and social values affect loyalty through their impact on satisfaction. Destination managers are encouraged to regularly evaluate how well the various aspects of perceived value are meeting the satisfaction levels of their tourists. Managers can utilize digital tools such as mobile apps, email, and websites to gather feedback on the perceived quality of the destination's offerings. By actively addressing the concerns and needs identified through these surveys, managers can enhance the overall service quality, leading to increased satisfaction and loyalty among tourists.

Thirdly, the research reveals gender-specific differences in how perceived value, satisfaction, and loyalty interrelate. Destination managers should be mindful of these differences and tailor their offerings to cater to the distinct preferences of male and female tourists. By providing a diverse range of activities and experiences, destinations can ensure that tourists from all genders can fully appreciate the value of the products and services offered, leading to a more inclusive and satisfying tourist experience.

## Limitations and prospects

While this study has laid a solid foundation for future research of this kind, it is not without its limitations, as is the case with many social science studies. Firstly, since the field investigation and data collection were concentrated in November and December, the research conclusions may be limited by the potential seasonal variations in demand. Secondly, this study examines the relationship between destination perceived value, satisfaction, and loyalty from the perspective of first-time visitors. There are significant differences in the travel motivations and experiences between first-time and repeat tourists in the tourism field. It is strongly recommended that future research compares differences among various visitor groups to better understand their distinctions. Thirdly, this study focused only on an open-pit mine ecological park, which limits the generalizability of the research findings. Future studies should conduct follow-up data surveys and storage for industrial heritage sites in different regions to verify the conclusions of this report. Fourthly, this study only assessed the moderating effect of gender on the research model. Future research should consider more other moderating factors, such as age, income level, and educational attainment.

## Data sharing agreement

The datasets used and/or analyzed during the current study are available from the corresponding author on reasonable request.

## Funding

This research was funded by.

1) Guangdong Philosophy and Social Science Planning Project (GD24XYS018)
2) Guangdong Philosophy and Social Science Planning Project (GD24XSH06)
3) 2023 Maoming Philosophy and Social Science Planning Project (2023YB18)
4) Projects of Talents Recruitment of GDUPT (2023rcyj2015).
5) Projects of PhDs' Start-up Research of GDUPT (2023bsqd1008) (2022bsqd2004)
6) Science and Technology Programme of Maoming of Guangdong Province of China (2023398), (2023411), (2024055)

## CRediT authorship contribution statement

**Nengjie Qiu:** Writing – original draft, Supervision, Project administration, Formal analysis, Data curation, Conceptualization. **Haibo Li:** Writing – original draft, Project administration, Methodology, Conceptualization. **Chen Pan:** Writing – original draft, Validation, Resources, Investigation. **Jiawei Wu:** Writing – review & editing, Visualization, Software. **Jiaming Guo:** Writing – review & editing, Resources, Investigation.

## Declaration of competing interest

The authors declare that they have no known competing financial interests or personal relationships that could have appeared to influence the work reported in this paper.
